# A case of patient with renal lupus with an initial presentation of hemolytic uremic syndrome triggered by streptococcal infection

**DOI:** 10.1002/ccr3.1425

**Published:** 2018-03-01

**Authors:** Rahaf Z. Attar, Enas I. Ramel, Osama Y. Safdar, Sherif Desoky

**Affiliations:** ^1^ College of medicine King Abdulaziz University Jeddah Saudi Arabia; ^2^ Faculty of Medicine Pediatric Nephrology Center of Excellence King Abdulaziz University Jeddah Saudi Arabia

**Keywords:** Hemolytic uremic syndrome, lupus nephritis, systemic lupus erythematosus, thrombotic microangiopathy

## Abstract

Systemic lupus erythematosus (SLE) is a systemic disease that is presented in a myriad of ways. Renal involvement is common in SLE and usually presents clinically as glomerulonephritis. We describe patients with SLE presented initially with hemolytic uremic syndrome which is a distinctive initial presentation.

## Introduction

Systemic lupus erythematosus (SLE) is a systemic autoimmune illness that can affect any organ or part of the body. The disease usually presents at an early age and has a median age of onset of 12–13 years. SLE is significantly more prevalent in females than in males, with a female‐to‐male ratio of 11:1 1. According to some studies, certain manifestations are more commonly found in early‐onset SLE, including malar rash, renal involvement, and hematological abnormalities such as hemolytic anemia and thrombocytopenia 2. The disease is diagnosed upon fulfillment of a certain number of clinical and laboratory criteria.

Renal involvement is common in patients with SLE. Indeed, several different patterns of renal disease are observed in patients with SLE, with immune complex‐mediated glomerular disease being the most common. Other patterns that present less frequently include tubulointerstitial and vascular diseases (e.g., thrombotic microangiopathy) 3, 4.

Hemolytic uremic syndrome (HUS) is a type of thrombotic microangiopathy defined by the presence of the classical triad of microangiopathic hemolytic anemia, thrombocytopenia, and acute kidney injury. The majority of cases are caused by the Shiga‐like toxin produced by *Escherichia coli*, and the presence of this toxin makes dysentery (bloody diarrhea) a common associated feature. This form of the disease is classified as typical HUS. Comparatively, the other type of HUS, which represents only a minority of cases, is not caused by the organism and moreover is not associated with bloody diarrhea. In fact, this type is linked to certain genetic abnormalities and complement dysregulation; hence, this type is called atypical HUS (aHUS) 5.

The literature contains very few reported cases of patients with SLE who first presented with HUS, and nearly all cases are associated with the atypical form. The several hypotheses regarding why such associations occur will be discussed later in this study.

In this report, we describe the case of a 9‐year‐old boy who presented with a history of pharyngitis and nonbloody diarrhea for a few weeks prior to admission; he was admitted to the hospital due to symptoms related to severe anemia and jaundice. The results of a laboratory work‐up and histopathological examination were consistent with thrombotic microangiopathy and stage IV lupus nephritis. Thus, a diagnosis of HUS secondary to SLE was established.

## Case Report

### Focused history

We report the case of a 9‐year‐old Saudi male who presented at King Abdulaziz University Hospital with a 3‐day history of pallor, lethargy, shortness of breath, and headache. Along with these manifestations, the family noticed a yellowish discoloration of the sclera. The patient was in optimal health, with no significant medical complaints, until 2 weeks prior to hospital admission, a time when he developed symptoms of upper respiratory tract infection (i.e., a sore throat, cough, and mild subjective fever). One week later, he developed diarrhea with a frequency greater than 5 times daily. The diarrhea was yellowish in color and without blood. Furthermore, the diarrhea was associated with epigastric pain and vomiting, and it resolved a few days later; however, the epigastric pain became more severe, and his vomiting continued. Due to this situation, the patient had been unable to maintain his usual appetite and had lost some weight. The patient also reported a decrease in the amount of his urine and a change of the urine's color, which was described by the mother as tea‐colored. No rash or edema was reported.

The patient was referred to our tertiary hospital by a dispensary after their results revealed red and granular casts in the urine and abnormal kidney function.

### Focused physical examination

An initial assessment revealed that the patient had a rapid but regular heart rate of 118 beats/min. His respiratory rate was 20/min, his blood pressure was 119/61 mmHg, and his oxygen saturation was 100%.

Examination showed that the patient was conscious and oriented; he was pale and had yellowish sclera but exhibited no other significant signs (i.e., no periorbital or lower limb edema, skin rash, or petechia). His chest was clear, and the heart sounds were normal, with no additional sounds or murmurs. A neurological examination was unremarkable.

Based on the initial assessment and the laboratory work‐up, (Table 1) hemolytic uremic syndrome was strongly suspected due to the presence of the classical triad of the disease. The diagnosis of PSGN was unlikely for several reasons, although the patient had a high ASO titer; this only confirmed a previous exposure and not an infection. Additionally, another more probable disease, i.e., HUS, better explained the abnormal kidney function and urine analysis. Unfortunately, we were unable to obtain a throat swab results and get an evidence of a streptococcal infection. Thus, PSGN could not be completely ruled out.

**Table 1:** Laboratory findings

Test	Results
CBC	Hb 4.8 g/dL [N: 12–14 g/dL], MCV 80.6 fL [N: 78–98 fL] platelet 128 × 10^9^/L [N: 150–400 × 10^9^/L], WBC: 13.9 10^9^/L [N: 4–11 10^9^/L], RBC 1.8 [N: 4.2–5.4 × 10^12^ M/μL], reticulocyte 11.61% [N: .5–1.5]
Blood film	Schistocyte +2, bite cells (+).
U&E	Urea 50.7 mmol/L [N: 2.5–6.4 mmol/L], creatinine 632 mmol/L [N: 53–115 μmol/L]
Urine dipstick	Hb +3, protein +2
Urine analysis	RBC 20/HPF [N: 0–10 RBC/HPF], blood 3 RBCs, albumin/creatinine ratio 2222.290 mg/g [N: 0–30 mg/g], protein ++ mg/dL
Immune profile	Antistreptolysin‐O (ASO) antibody 533 IU/mL [N: 0–200 IU/mL], C3 0.45 g/L [N: 0.75–1.65 g/L], anti‐C4 antibody 0.15 [N: 0.2–0.6 g/L], antinuclear antibody (ANA) 1:320 moderate positive, anti‐double‐stranded DNA (dsDNA) 247.6 IU/mL [N: 0–200 IU/mL], anticardiolipin antibody‐M: 1.6 U/mL [N: 0–7 U/mL], anti‐B2‐glycoprotein IgG: 0.08 U/mL [N: 0–10 U/mL]
Coombs test	Negative
Throat swab	Unavailable results due to improper specimen
Stool analysis culture	Negative for verotoxin‐producing organisms, and no RBCs were detected.
Other tests	LDH: 1610 IU/L [N: 105–333 IU/L], haptoglobin < 0.3 g/L [N: 0.5–3.2 g/L], CRP: 8 mg/L [N: 0–3 mg/L], serum albumin 27 g/L [N: 40.2–47.6 g/L]

CBC, complete blood count; CRP, C‐Reactive protein; Hb, hemoglobin; LDH, lactate dehydrogenase; MCV, mean cell volume; RBC, red blood cells; WBC, white blood cells.

There are few similar cases reported in the literature involving patients with SLE who initially present with HUS 10, 11, 12, 13, 14, 15. (Table 2)

**Table 2:** Summary of patients with SLE with initial presentations of aHUS

Author	Year	Patient	Histological diagnosis and laboratory results	Management
Morioka et al. 10	1995	24‐year‐old female	Severe fibrinoid necrosis of the arterioles, mainly in the glomerular afferent arteriole associated with diffuse proliferative lupus nephritis	Methylprednisolone pulse therapy, hemodialysis, and double filtration plasmapheresis were performed.
Ogawa et al. 11	2000	10‐year‐old female	Picture of thrombotic microangiopathy	Methylprednisolone
				Cyclophosphamide
				Plasma exchange
Tsao et al. 12	2002	13‐year‐old male	Picture of thrombotic microangiopathy	Methylprednisolone cyclophosphamide pulse therapies
				No plasmapheresis or plasma infusion was required.
Kawasaki et al. 13	2002	12‐year‐old female	Picture of thrombotic microangiopathy	Methylprednisolone cyclophosphamide
				No plasmapheresis or plasma infusion was required.
Rabbani et al. 14	2005	21‐year‐old female	All glomeruli displayed diffuse proliferation. Arterioles had fibrinoid necrosis or thrombotic luminal occlusion.	Methylprednisolone, cyclophosphamide and plasmapheresis therapies
Azharuddin et al. 15	2005	25‐year‐old female	Picture of thrombotic microangiopathy	Steroids, plasmapheresis with cryosupernatant and cyclophosphamide

Surprisingly, the patient also exhibited a positive immune profile for SLE (i.e., ANA and dsDNA).So far, a clear diagnosis of the acute kidney injury was difficult to be made. For this reason, a kidney biopsy was indicated.

## Histopathology

### Light microscopy

**Figure 1:**
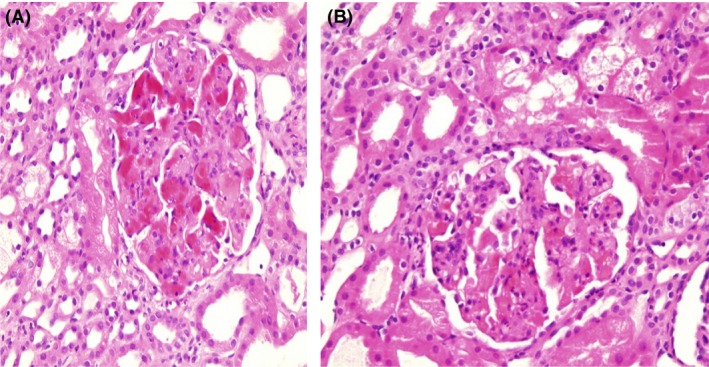
(a, b) Marked glomerular congestion with distention of the capillary lumen in addition to segmental fibrinoid necrosis.

**Figure 2:**
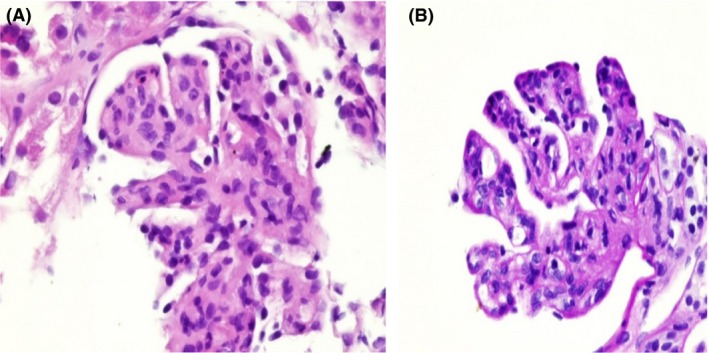
(a, b) Membranoproliferative pattern: mesangial matrix expansion, mesangial and endocapillary proliferation. Diffuse thickening of the glomerular basement membrane (GBM). Segmental intracapillary nucleated RBCs in some capillary loops. Segmental mesangiolysis.

**Figure 3:**
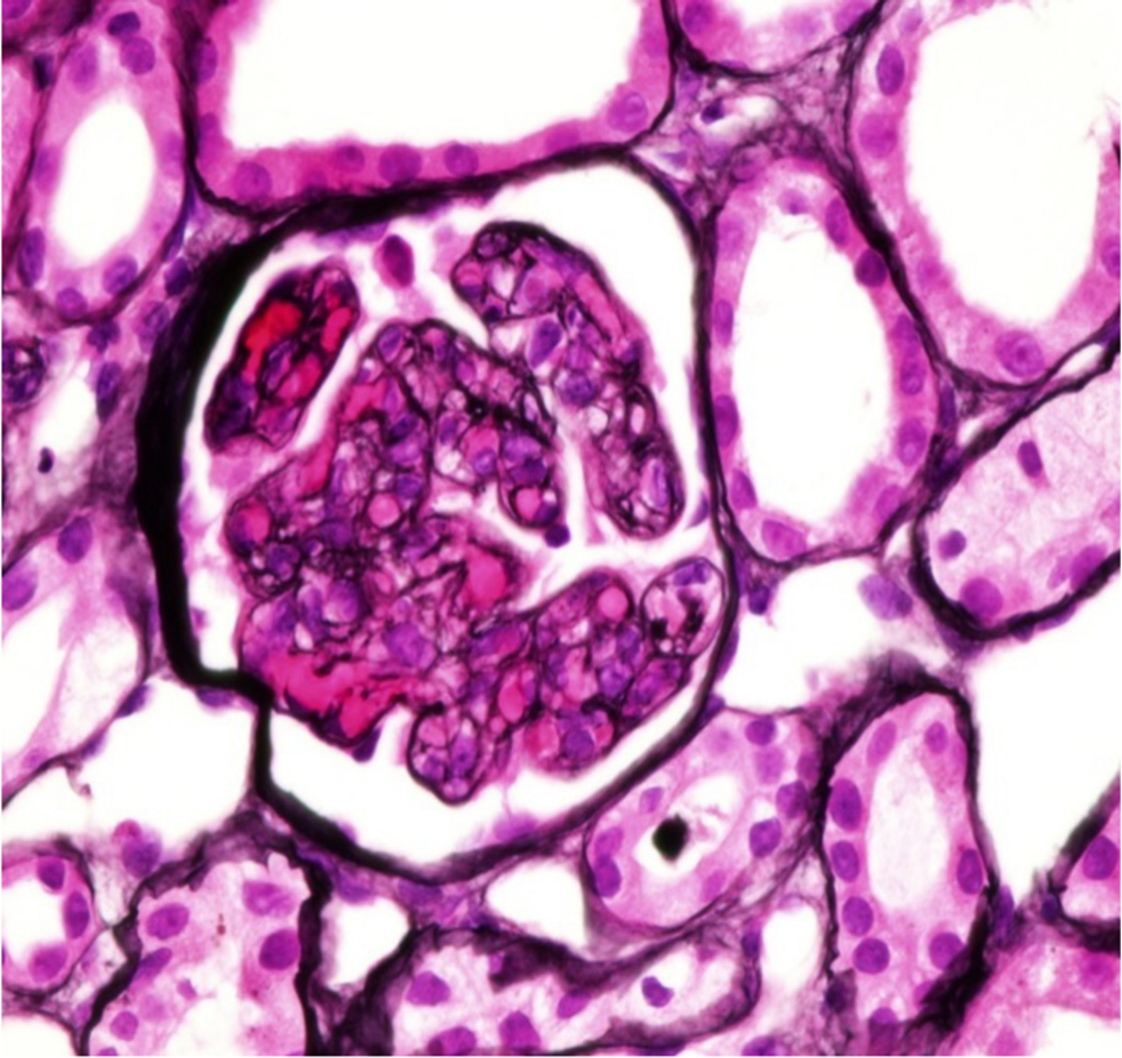
Segmental double contour of the glomerular basement membrane.

Light microscopic examination of 33 glomeruli revealed two distinctive patterns. A glomerular disease pattern was observed that involved diffuse thickening and a double contour of the glomerular basement membrane with severe mesangial expansions, segmental endocapillary proliferation of 13 glomeruli, and a few other glomeruli with membranoproliferative patterns. Additionally, a few glomeruli exhibited cellular crescent formations. Marked glomerular congestion with intracapillary leukocyte infiltration was present in nearly half of the glomeruli, and moderate edema of the interstitium with mild mixed inflammation and no fibrosis was also present (Fig. 1, 2, 3).

**Figure 4:**
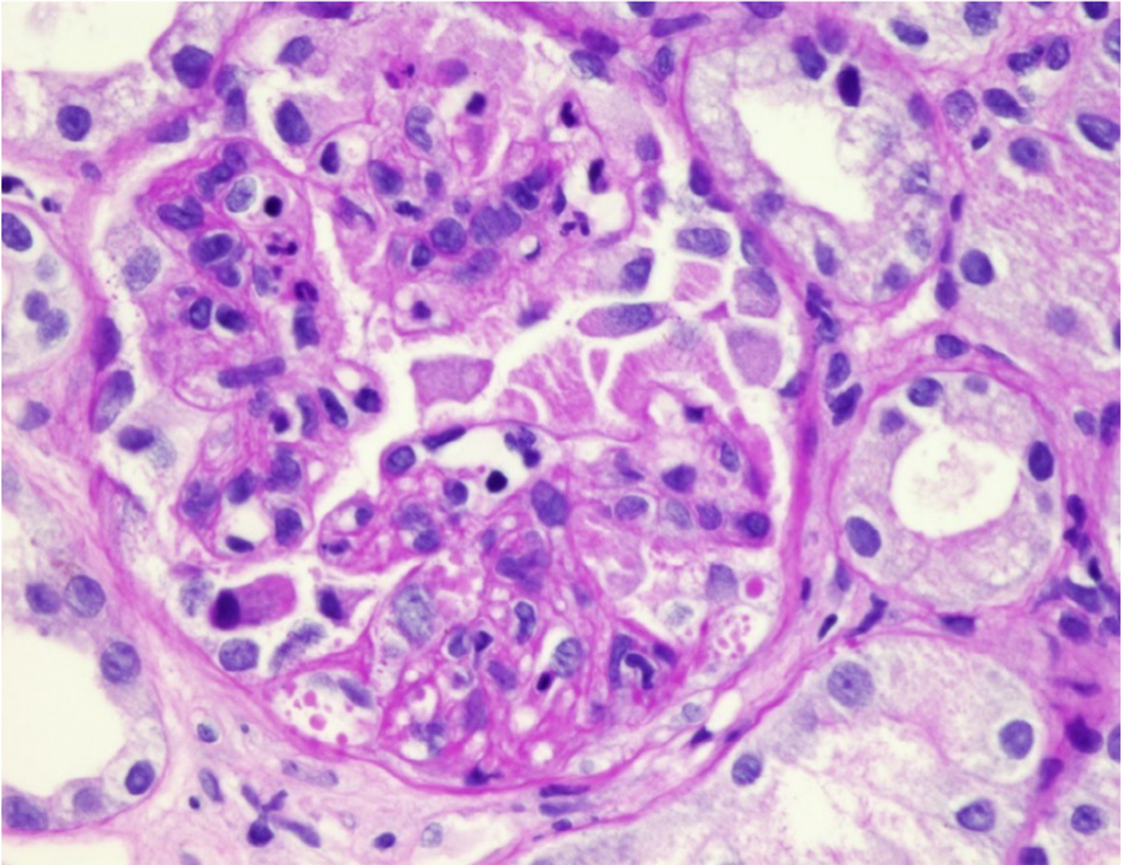
Segmental fibrinoid necrosis.

**Figure 5:**
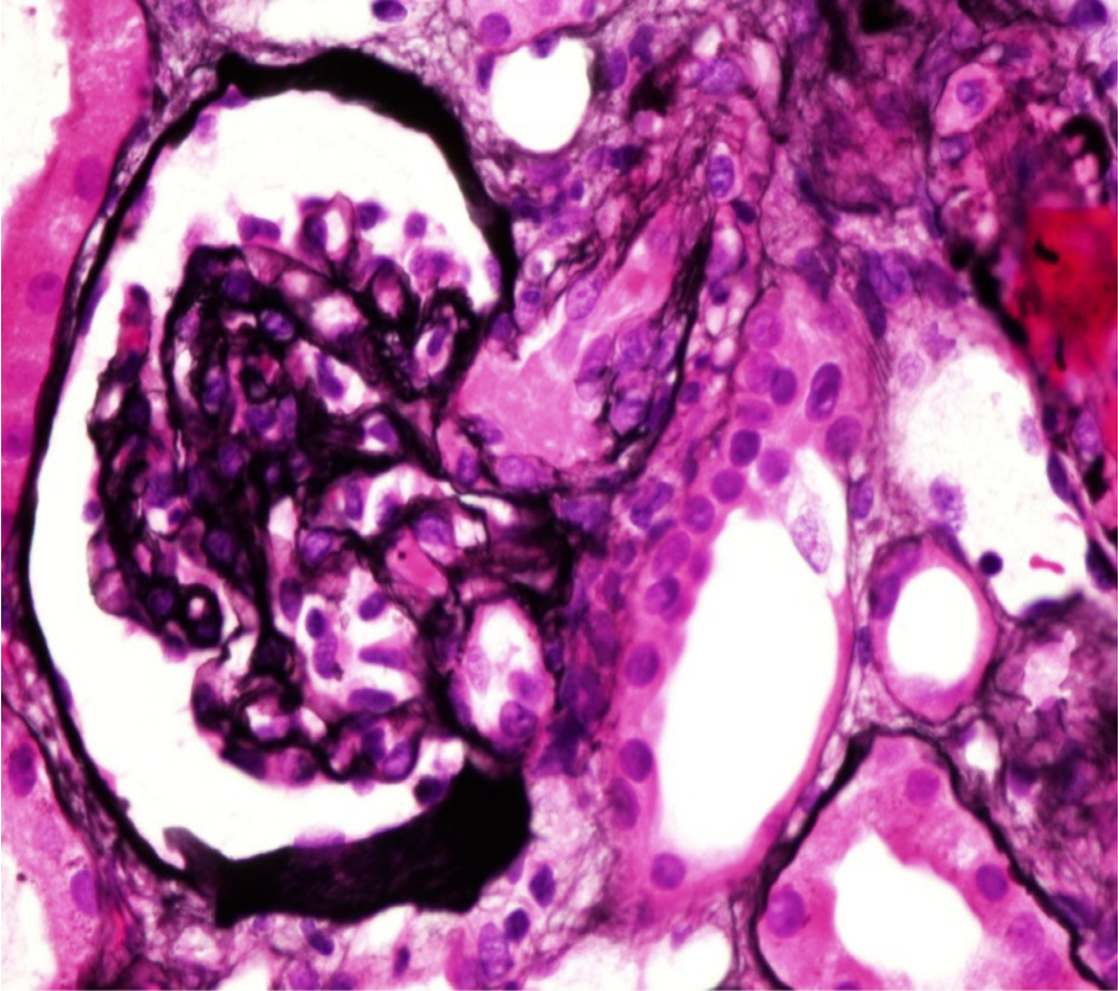
Hilar arteriole exhibiting intimal fibrinoid necrosis occluding the lumen.

**Figure 6:**
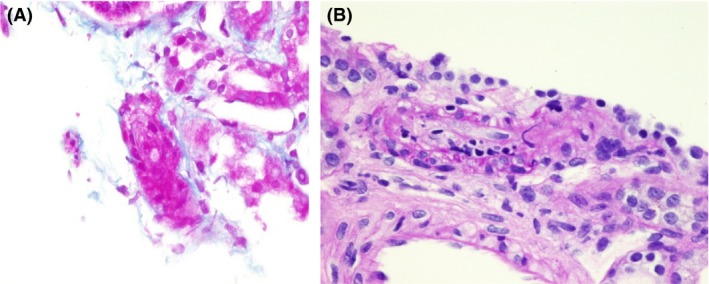
(a, b) Small artery exhibiting intimal fibrinoid necrosis and concentric fibrointimal hyperplasia occluding the lumen, with intimal leukocytes in the wall.

A microangiopathic occlusive pattern was noted. Some of the glomeruli exhibited global ischemic collapse with marked glomerular congestion. Several hilar arterioles exhibited fibrinoid necrosis occluding the lumina with intraluminal fibrin thrombi and concentric fibrointimal hyperplasia (Fig. 4, 5, 6).

### Immunofluorescence study

We observed +3 coarse granular positivity along with the capillary loop and mesangium for C3 and IgG and +2 for IgM (Fig. 5), with trace positivity for C1q and negative staining for C4.

### Final diagnosis

The diffuse proliferative sclerosing pattern that was observed along with early crescent formation and the positive results for ANA and anti‐dsDNA antibodies confirmed the diagnosis of stage IV lupus nephritis according to Systemic Lupus Collaborating Clinics (SLICC) criteria. 6

Additionally, the microangiopathic picture, which included the presence of fibrin thrombi occluding the lumina and global ischemic collapse, confirmed HUS. The patient likely had an atypical form of the disease because no organisms or blood were detected in his stool. The presence of SLE supports this diagnosis because SLE is known to be a secondary cause of aHUS.

### Hospital course

Initially, the patient received supportive treatment for his kidney injury and packed RBCs for his severe anemia. Following local guidelines, the patient was given six IV doses of methylprednisolone pulses, i.e., 600 mg/m^2^ per dose, due to our initial suspicion of rapidly progressive glomerulonephritis and eventually to minimize his organ damage and control his SLE flare.

During his hospital stay, the patient started to develop edema and hypertension. He required dialysis three times, and an additional three blood transfusions. Plasma exchange was not available at our hospital at that time. Fortunately, the patient had improved on the supportive management given and did not need plasma exchange or transfusion.

Eventually, he was discharged home after his kidney function was normalized with both 1 mg/kg of prednisone and monthly injections of 1 mg/m^2^ of cyclophosphamide for 5 months.

### Follow‐up

No follow‐up information or repeated tests were obtained as the patient's family decided to continue his management in a different hospital.

## Discussion

SLE is an autoimmune multisystemic disease that has a myriad of clinical presentations. One or two organs are usually initially involved, and other organs might be involved after a certain period of time. A French multicenter study 7 that included 155 pediatric patients with SLE demonstrated that the most commonly involved systems in the initial presentation of SLE are the hematological and musculoskeletal systems, with percentages of 72% and 64%, respectively. Renal involvement is less common and occurs in only 50% of the pediatric population compared with 23% of adults 8. Half of all patients with SLE with kidney disease have a clinically evident disease that presents with symptoms of acute kidney injury, chronic renal failure, nephrotic and nephritic syndromes; the other half presents with subclinical diseases such as an asymptomatic elevation in creatinine, abnormal urine analysis, or kidney pathology 9. The present case is interesting because of the unusual and distinctive presentation of lupus nephritis a thrombotic microangiopathy.

All of these cases were associated with aHUS. There are several hypotheses regarding why such an association would occur. Antiphospholipid antibodies, complement dysregulation, and infections have been suggested to play roles in the pathogenesis of thrombotic microangiopathy in patients with SLE.

The literature includes several reported SLE cases with renal thrombotic microangiopathy significantly associated with the presence of antiphospholipid antibodies 10, 16, 17. For example, Bridoux et al. 16. evaluated eight patients with SLE with renal thrombotic microangiopathy, and antiphospholipid antibodies were found in five of these patients. This finding reveals the significant association between antiphospholipid antibodies and the thrombotic microangiopathic complications of SLE. However, the presence of HUS/thrombotic thrombocytopenic purpura (TTP) was only established in one patient. This hypothesis is not relevant to our case because our patient had unremarkable antiphospholipid antibody levels. Nonetheless, further assessment of this association is needed.

Genetic defects and complement dysregulation are important suggested etiologies of aHUS. In fact, it has been estimated that at least 50% of patients with aHUS have genetic mutations for the following complement proteins: Factor H, I, and B, and CD46 18, 19. Factor H (i.e., a complement regulator of the alternative complement pathway) in particular was found to be correlated with both SLE and aHUS. Recent studies have demonstrated that people with factor H deficiencies are susceptible to SLE. Moreover, such deficiency accelerates the development of lupus nephritis 20. Other studies have also implicated low factor H levels in the pathogenesis of aHUS, causing thrombotic microangiopathy due to endothelial injury and platelet consumption 21.

Infection is another factor that has been correlated with the occurrence of thrombotic microangiopathy in patients with SLE. Ming‐Han Chen et al. 22. evaluated 25 patients with SLE who developed thrombotic microangiopathy either after or simultaneous with the diagnosis of SLE, revealing association with infections in 16 of the 25 patients (64%).

Because our patient complained of pharyngitis and presented with prodromal diarrhea before his hospital admission, we believe that an infection might have been the trigger in this case. However, we do not have any valid evidence of an infection to support our claim.

Conditions other than HUS have been associated with thrombotic microangiopathy in SLE, including TTP, catastrophic antiphospholipid syndrome, malignant hypertension, and scleroderma 23.

The presentations of HUS and TTP are very similar 24. If the occlusive thrombotic pattern is associated with the classical clinical triad (i.e., renal failure, thrombocytopenia, and microangiopathic hemolytic anemia), the diagnosis of HUS is more likely, as previously discussed. In contrast, TTP usually manifests with a pentad that includes the same symptoms as HUS in addition to neurological deficit and fever, both of which were absent in our patient. However, half of patients with TTP lack one or more of these classical symptoms, which makes it difficult to clinically distinguish between the diseases, particularly when the suspected HUS is atypical and no organism or toxin can be identified. Several studies have established that vWF‐cleaving protease activity is typically low in TTP cases due to a defect in the ADAMTS13 gene, whereas this level is normal in HUS. This single parameter can be used to distinguish between the diseases when needed 25.

In general, renal thrombotic microangiopathy when associated with SLE indicates a serious complication that increases morbidity and mortality.

Plasmapheresis is one measure that has been demonstrated to remarkably increase the remission rate and decrease the mortality of these patients as well as any patient with aHUS in general. The established mortality rate for plasma exchange‐treated patients is 25%, and that for untreated patients is 57% 26, 27.

Response to plasma exchange in patients with aHUS depends on the type of complement deficiency. Two‐thirds of patients with complement H and I deficiencies experience remission from the disease. Other types are more likely to recur 28.

Nonetheless, all reported cases in which HUS was implicated as an initial presentation of SLE improved following treatment with prednisolone and an immunosuppressive agent with or without plasmapheresis or infusion. This indicates that the outcome of aHUS in SLE is generally favorable.

Eculizumab, a monoclonal antibody against C5 is a newer treatment option for patients with aHUS. It has the same efficacy in patients with and without known mutations. Studies to date have demonstrated that eculizumab normalizes renal function in aHUS patients and decreases the frequency of renal dialysis. It is even effective in patients who are refractory to plasma exchange and patients who have undergone renal transplantation 29, 30.

Our patient was also treated with prednisone and cyclophosphamide, but no plasmapheresis or plasma infusion was required. Most children with hemolytic uremic syndrome recover after conservative management. Moreover, plasma exchange is generally reserved for more severe and atypical cases.

## Conclusion

There are several reported cases in the literature of patients with SLE who first present with thrombotic microangiopathy. Antiphospholipid antibodies, infections, and complement dysregulation have been suggested to play roles in these cases. Despite all of these associations, the underlying pathophysiology remains unknown, and further studies are required. The outcomes of aHUS in patients with SLE are generally favorable.

## Conflict of Interest

None declared.

## Authorship

RA: First author, reviewed literature wrote the abstract & discussion. OS: corresponding Co‐author, reviewed the manuscript. ER: co‐author, wrote the clinical presentation and references. SD: Senior co‐author, reviewed the manuscript.
